# Predicting HER2 Status in Breast Cancer on Ultrasound Images Using Deep Learning Method

**DOI:** 10.3389/fonc.2022.829041

**Published:** 2022-02-16

**Authors:** Zilong Xu, Qiwei Yang, Minghao Li, Jiabing Gu, Changping Du, Yang Chen, Baosheng Li

**Affiliations:** ^1^ Laboratory of Image Science and Technology, School of Computer Science and Engineering, Southeast University, Nanjing, China; ^2^ Laboratory of Radiation Oncology, School of Medicine, Shandong University, Jinan, China; ^3^ Department of Radiation Oncology, Shandong Cancer Hospital and Institute, Shandong First Medical University and Shandong Academy of Medical Sciences, Jinan, China

**Keywords:** breast cancer, ultrasound, deep learning, DenseNet, human epidermal growth factor receptor 2

## Abstract

**Purpose:**

The expression of human epidermal growth factor receptor 2 (HER2) in breast cancer is critical in the treatment with targeted therapy. A 3-block-DenseNet-based deep learning model was developed to predict the expression of HER2 in breast cancer by ultrasound images.

**Methods:**

The data from 144 breast cancer patients with preoperative ultrasound images and clinical information were retrospectively collected from the Shandong Province Tumor Hospital. An end-to-end 3-block-DenseNet deep learning classifier was built to predict the expression of human epidermal growth factor receptor 2 by ultrasound images. The patients were randomly divided into a training (n = 108) and a validation set (n = 36).

**Results:**

Our proposed deep learning model achieved an encouraging predictive performance in the training set (accuracy = 85.79%, AUC = 0.87) and the validation set (accuracy = 80.56%, AUC = 0.84). The effectiveness of our model significantly exceeded the clinical model and the radiomics model. The score of the proposed model showed significant differences between HER2-positive and -negative expression (*p <* 0.001).

**Conclusions:**

These results demonstrate that ultrasound images are predictive of HER2 expression through a deep learning classifier. Our method provides a non-invasive, simple, and feasible method for the prediction of HER2 expression without the manual delineation of the regions of interest (ROI). The performance of our deep learning model significantly exceeded the traditional texture analysis based on the radiomics model.

## Introduction

Human epidermal growth factor receptor 2 (HER2) is an important biomarker and a target in the therapy used in approximately 30% of breast cancer patients (1, 2). Although HER2-enriched cancers may have a worse prognosis, they can be effectively treated with therapies targeting HER2 protein, such as Herceptin (chemical name: trastuzumab), Perjeta (chemical name: pertuzumab), and Kadcyla (chemical name: T-DM1 or ado-trastuzumab emtansine) (3). Breast cancer molecular subtypes are categorized in clinical practice by immunohistochemical markers.

The recent literature shows that radiomics features extracted from medical images may predict patient outcomes (4–6). Breast cancer diagnosis in clinical practice is performed using a type of radiation-free medical imaging approach, and ultrasound imaging plays a significant role (7–10). The association of peritumoral radiomics features extracted from magnetic resonance imaging (MRI) and the expression of HER2 was established (11).

In recent years, besides the development of compressed sensing (12, 13), wavelet transform (14), and dictionary learning techniques (15–17), deep learning approaches have become popular in the field of medical image processing with the development of optimization techniques and the improvement in computational devices (18). The deep learning method-based classification has a positive impact in precision medicine, since it can improve the effectiveness of computer-assisted clinical and radiological decision (19). Existing literature describes the use of the deep learning method to predict medical targets, such as EGFR mutation status in lung cancer (20), and recurrence in high-grade serous ovarian cancer (21, 22).

Deep learning automatically generates the representations that are expressed in terms of other, simpler representations through gradient descent and back-propagation. The abstract mapping from the raw data to the target label is built as a training result (23). DenseNets developed for image tasks have several advantages: avoid the vanishing-gradient, reuse features, and reduce the number of parameters (24). DenseNet (24) exceeds AlexNet (25), GoogLeNet (26), VGG (27), and ResNet (28) in the ImageNet classification task.

In this study, a dense-block-based deep learning model was developed to predict HER2 expression based on preoperative ultrasound images. This proposed method like other supervised deep learning models is an end-to-end workflow. The model requires only a rectangle region of the tumor without the precise delineation of the tumor boundary or human-defined features, while conventional radiomics methods depend on feature engineering. The interobserver error can be reduced and the time for manual segmentation can be saved through our method. The proposed deep learning model can automatically learn HER2 expression features from ultrasound images through back-propagation and optimization algorithm (23). An ultrasound image dataset collected from the Shandong Cancer Hospital and Institute was provided to train and evaluate our deep learning model.

## Material and Methods

**Figure 1:**
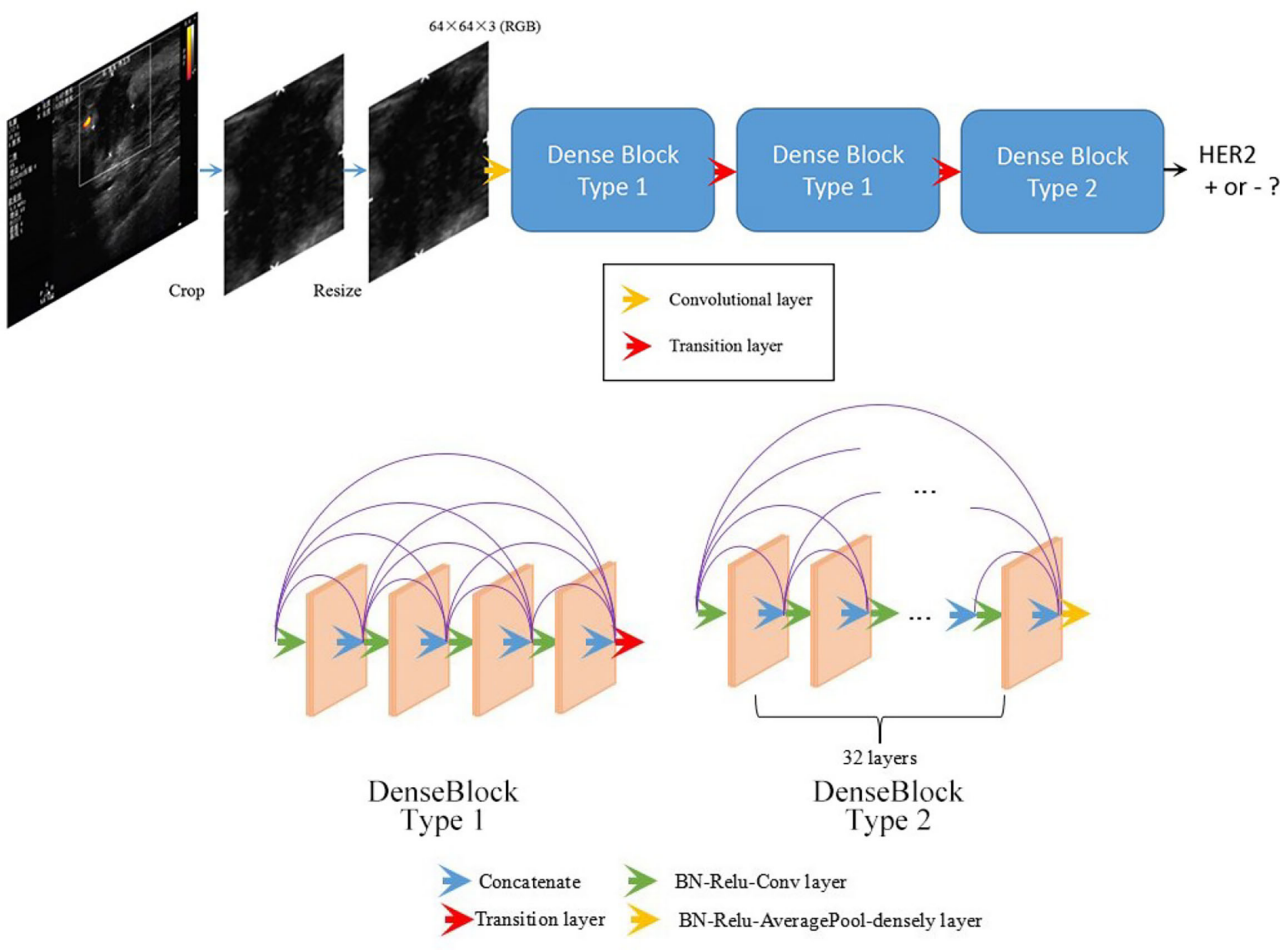
Overall structure of the developed DenseNet-based deep learning classifier. Being fed a tumor image, the deep learning model predicts the probability of the expression of HER2.

This work used a DenseNet-based deep learning model to predict breast cancer molecular subtypes from the ultrasound images. The workflow is shown in **Figure 1**.

### Patients

This retrospective study was approved by the ethics review board. Preoperative ultrasound images of 144 patients were collect from the Shandong Cancer Hospital and Institute. The collected ultrasound images were obtained by an experienced radiologist using a broadband 42–46-Hz transducer (Philips Healthcare System, Amsterdam, Netherlands). Most of the images were cross-section images, the remaining were longitudinal sections. For consistency, only cross-section ultrasound images were used. The whole dataset was randomly divided into a training set and a validation set through the hold-out method. The training set and the validation set were mutually exclusive.

In clinical practice, the molecular subtypes can suggest candidate drugs for the treatment of these patients (29). Immunohistochemistry (IHC) is the most common clinical approach for immunostaining. Since IHC can accurately identify the molecular subtypes of breast cancer by high specificity, in this work, the molecular subtype were identified by IHC. The resulting score of 0, 1+, 2+, and 3+ in the IHC staining represented the amount of HER2 protein on the surface of the cells in a breast cancer tissue sample. The score 0 or 1+ indicate “HER2 negative.” The score 2+ is called “borderline.” The score 3+ indicates “HER2 positive.” If the result of IHC is uncertain, the fluorescence *in situ* hybridization (FISH) was carried out.

Our inclusion criteria of the data were as follows: (1) pathologically confirmed breast cancer; (2) available preoperative ultrasound image data; (3) pathological IHC examination of tumor specimens; and (4) no history of preoperative therapy. Our exclusion criteria were as follows: (1) ultrasound images were too ambiguous to be analyzed and (2) invasive biopsy was performed before the ultrasound examination.

A rectangle region of interest (ROI) containing the entire tumor was manually selected by radiologists. The ROI was enough due to the strong capability of the information extraction of the deep learning model. Consequently, the precise drawing of the tumor border was not necessary.

### Development of the Deep Learning Model

In comparison with previous popular network architectures, DenseNets leverage shortcut connections enhance the information flow to provide better effectiveness. The shortcut connection can be defined as follows:


(1) $$x_{k}=H_{k}([x_{0},\;x_{1},\ldots ,\;x_{k-1}])$$

where [*x*_0_ *, x*_1_ *,…, x_k_*_−1_] refers to the concatenation of the deduced feature maps in the *k*th layers (24).

Before training the network, several data preprocessing procedures were carried out: ROI selection, image cropping, and image resizing. In each ultrasound image, a rectangle region containing the whole tumor and the tumor borderline was selected. Although tumors have different sizes, all ROI including tumors were scaled to the same size (64 × 64 pixels) by bilinear interpolation before being fed into the network.

The structure of the model was designed in an attempt to achieve better results. Our proposed network was composed of three dense blocks. Two types of dense block were present among these three dense blocks (see **Figure 1**) in our network. Block type 1 contained 4 layers, while block type 2 contained 32 layers. Both the two types of dense block employed shortcut connections from each layer to all subsequent layers. The details of the two types of dense blocks are shown in **Figure 1**. The detailed structure of the entire network is indicated in **Supplementary Table 1**.

The deep learning model was implemented based on the TensorFlow (30) framework and Python 3.5 (31). The trained model gradually becomes stable as the batch size increases, resulting in less overfitting. The weighting coefficient for the classification was adjusted for the imbalance of the classes. Weighting cross-entropy was used as the loss function in our implementation. This approach could help us avoid downsampling or upsampling of the original data; thus, our data distribution was close to the real clinical data. The weight coefficient was tuned, and then a series of experiments were performed. The best configuration was related to the label distribution of the training data. The detailed parameter setting for training the model is indicated in **Supplementary Table 2**.

### Visual Analysis of the Model

The shallow convolutional layer learned low-level simple features such as the horizontal and diagonal edges. A deeper convolutional layer learned more complex features such as tumor shape. The features learned by the low-level layers were intuitive, while the learned features became more abstract with the layers deepening and could gradually be related to the molecular subtypes.

**Figure 2:**
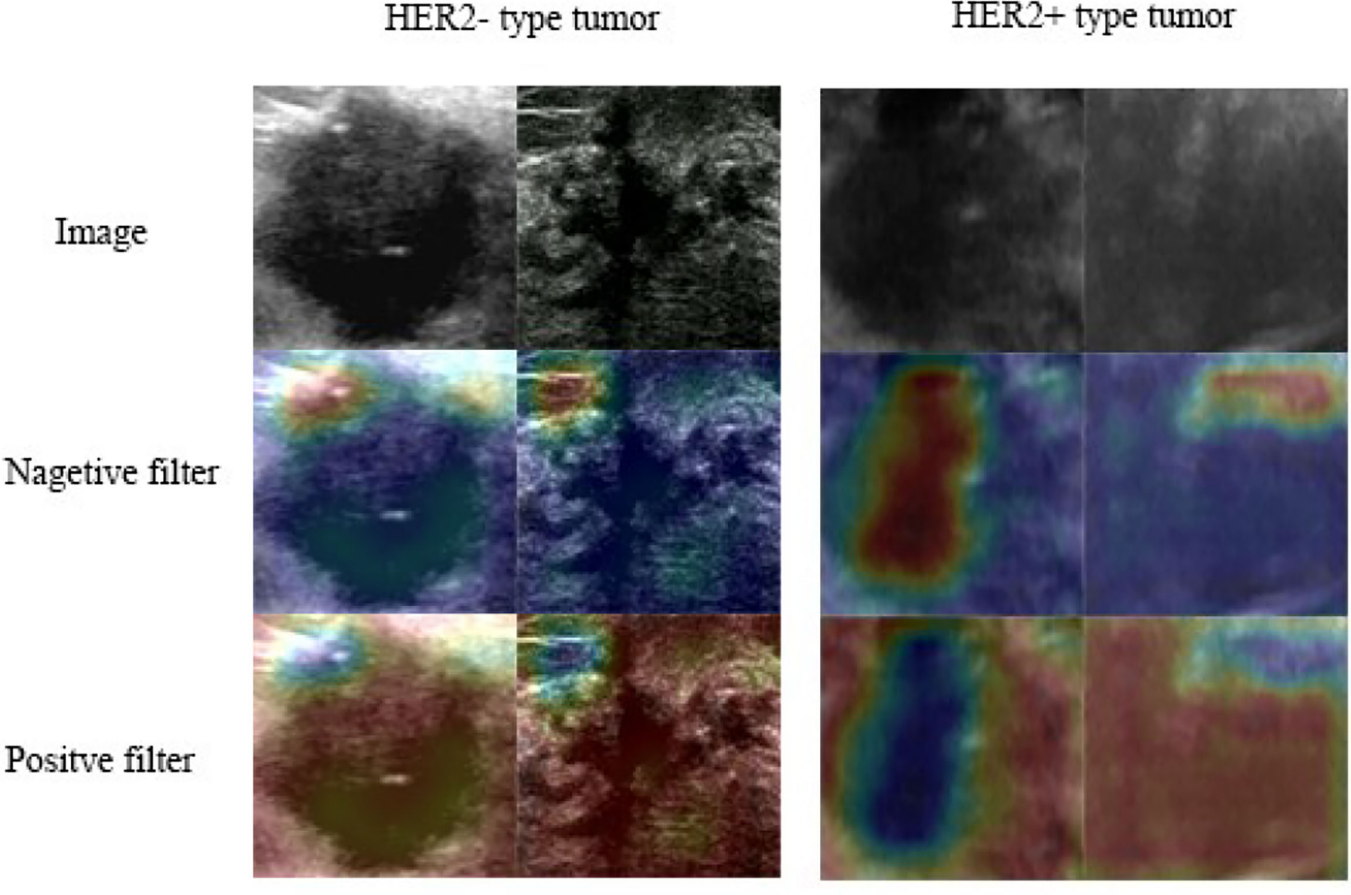
Class activation heat map: the attention map of the trained model for predicting HER2 expression.

The class activation map method was used to generate an attention map of the trained model for visualization (32, 33). This method helped to visualize and highlight the discriminative image parts detected by the feature extractor, which contributed to the predicted class scores on any given image. The examples of attention map are shown in **Figure 2**. The positive filter tended to focus on the boundary of the tumor or the high echo region. In the HER2 case, the positive filter indicated the HER2+ category, while the negative filter corresponded to the HER2- category. The positive filter needed to collect more information from a larger area to make a decision than the negative filter.

### Statistical Analysis

Statistical analysis was performed using a Python language-based toolkit including SciPy (34), scikit-learn (35), and WORC packages. The Mann–Whitney U-test (36) was used to compare the age difference, while a chi-square test (37) was used to compare the difference in other factors. The DeLong test was used to evaluate the difference of the receiver operating characteristic (ROC) curves among different models. A *p*-value *<* 0.05 was considered statistically significant.

## Results

### Clinical Characteristics of the Patients

The clinical characteristics of the patients are listed in **Table 1**. No significant difference was found between the training and validation cohorts in terms of age, TNM stages, and BI-RADS. These clinical characteristics were also used to build a clinical model for the comparison with the proposed deep learning model.

**Table 1:** Clinical characteristics of patients in the primary and validation cohorts.

Factors	Total	Testing cohort	Training cohort	*p*-value
Subjects n	144	108	36	
Age (years)	53.5 ± 10.6	40 ± 11.3	49 ± 7	0.535
T stage				
T1	55	47 (44.1)	8 (22.2)	
T2	82	54 (50)	28 (77.8)	0.361
T3	4	4 (2.9)	0 (0)	
T4	3	3 (2.8)	0 (0)	
N stage				
N1	77	63 (57.9)	14 (38.4)	
N2	41	28 (26.3)	13 (38.3)	0.236
N3	23	17 (15.8)	6 (15.4)	
M stage				
M0	138	105 (97.3)	33 (91.0)	
M1	6	3 (2.6)	3 (9.0)	0.337
Total stage				
I	44	31 (29.6)	11 (30.0)	
II	70	52 (48.6)	18 (50.0)	0.347
III	32	25 (22.9)	7 (20.0)	
IV	1	1 (0.9)	0 (0)	
BI-RADS				
III	11 (7.6)	7 (6.5)	4 (11.1)	
IV	100 (69.4)	79 (73.2)	21 (58.3)	0.718
V	33 (22.0)	22 (20.3)	11 (30.6)	

(1) Data are presented as mean ± SD, or n (%) unless otherwise stated.

(2) The Mann–Whitney U-test was used to compare the age difference. The chi-square test was used to compare the difference in other clinical factors.

### Prediction Performance of the Proposed Deep Learning Model

**Figure 3:**
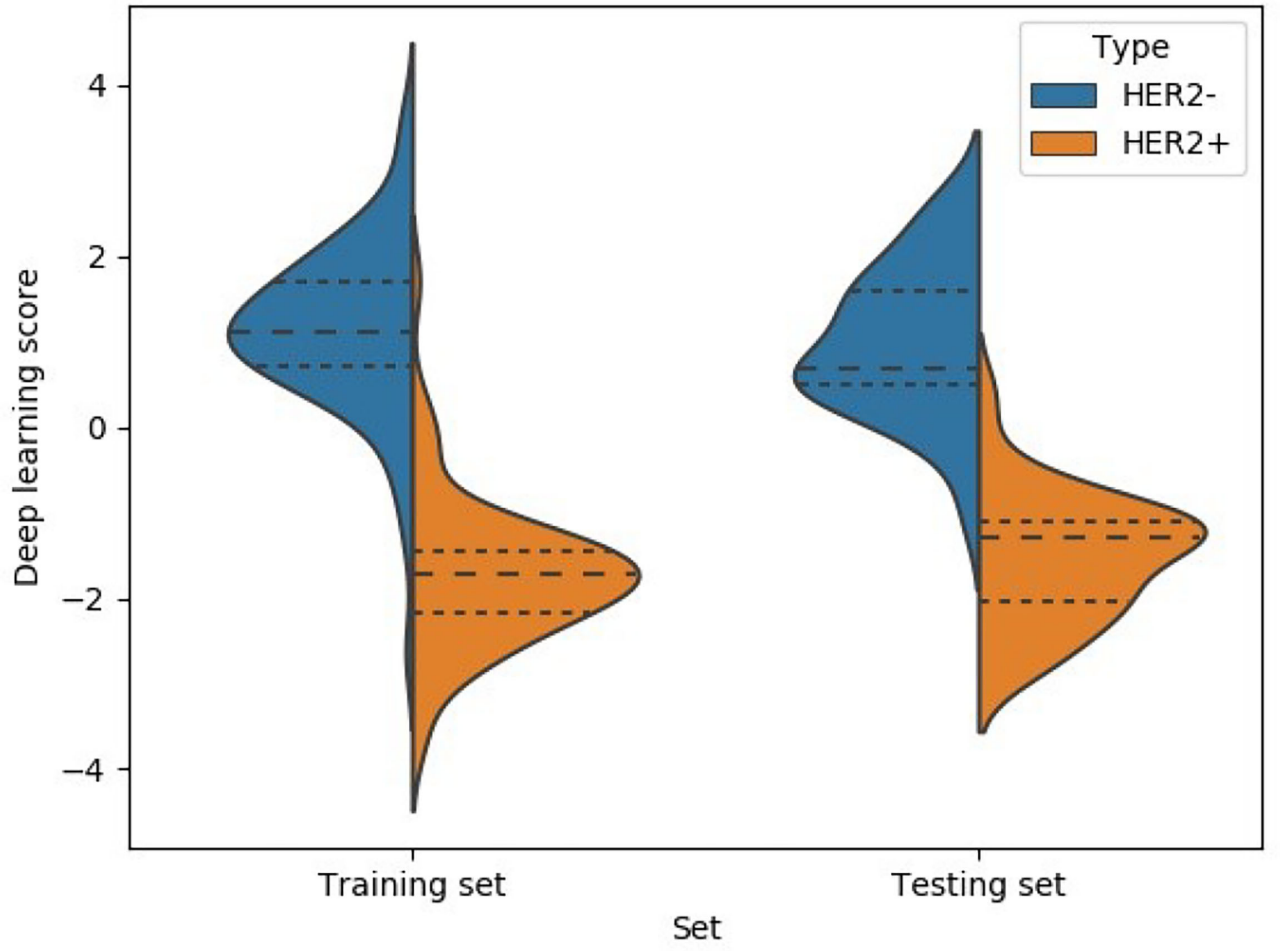
Deep learning model score HER2 classifier.

**Figure 4:**
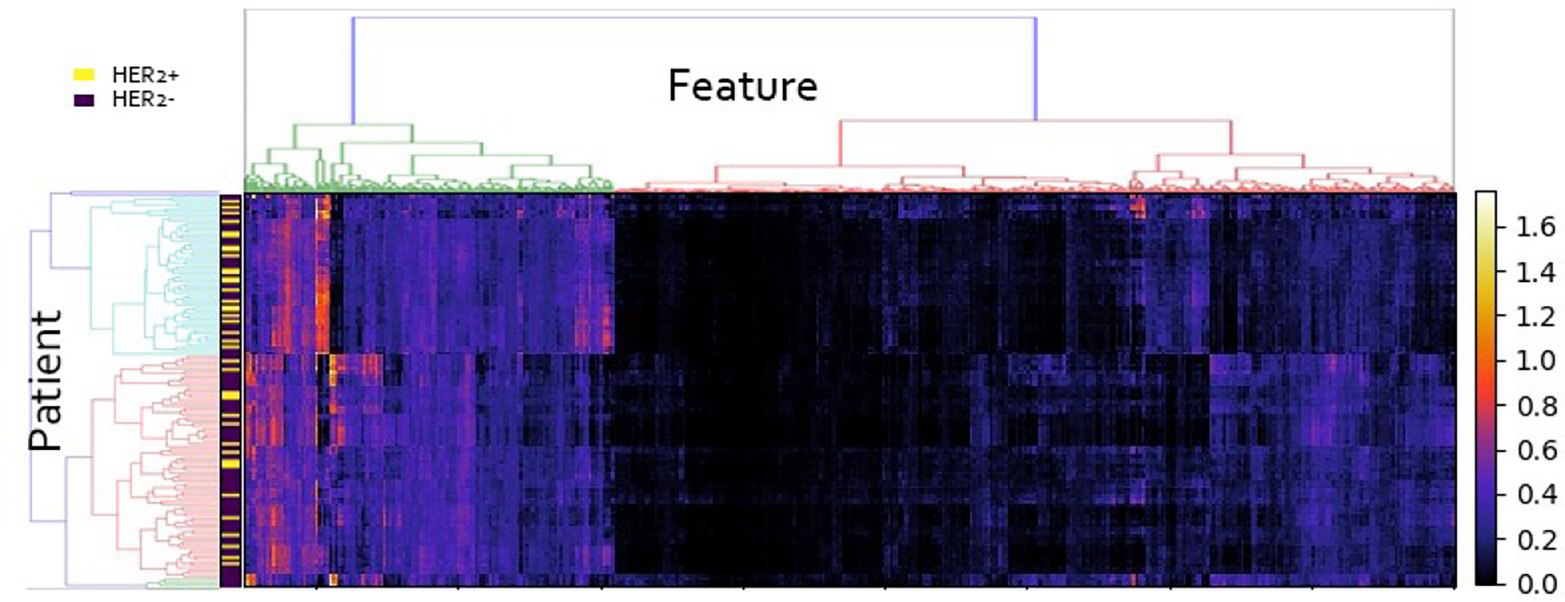
Cluster analysis of deep learning features.

A 3-dense-block-based deep learning model using preoperative ultrasound images was proposed in this study to predict HER2 expression in patients with breast cancer. Our deep learning model showed promising results of accurate predictions. The DL model achieved an AUC of 0.87 in the training cohort (accuracy = 85.19%, sensitivity = 75.53%, specificity = 90.54%, PPV = 78.12%, NPV = 88.16%) and AUC of 0.84 in the validation cohort (accuracy = 80.56%, sensitivity = 72.73%, specificity = 84.00%, PPV = 66.67%, NPV = 87.5%). The result of the experiment allowed us to conclude that the performance of the deep learning model significantly exceeded the traditional radiomics model. Moreover, the deep learning score between HER2+ and HER2- type groups in the training cohort and validation cohort was significantly different (*p <* 0.01; **Figure 3**). A radiomics model was also built for comparison to predict the Luminal type. The PyRadiomics toolkit was used to extract image features, and then six features were selected by the recursive feature elimination. Finally, a random forest including 90 trees was built in the radiomics model for prediction. Deep learning features were extracted from the last convolutional layer (global average pool) for cluster analysis (see **Figure 4**). The clustering figure suggested that the deep learning features have different responses to positive and negative cases.

### Comparison Between the Deep Learning Model and Other Methods

A clinical model and a radiomics model were built as a comparison to the proposed deep learning model. The clinical model considered age, stage, and BI-RADS as features and employed a support vector machine as the classifier. In the radiomics model, 961 features were extracted through the PyRadiomics toolkit. A random forest classifier was built for the prediction of HER2 expression in the radiomics model.

**Figure 5:**
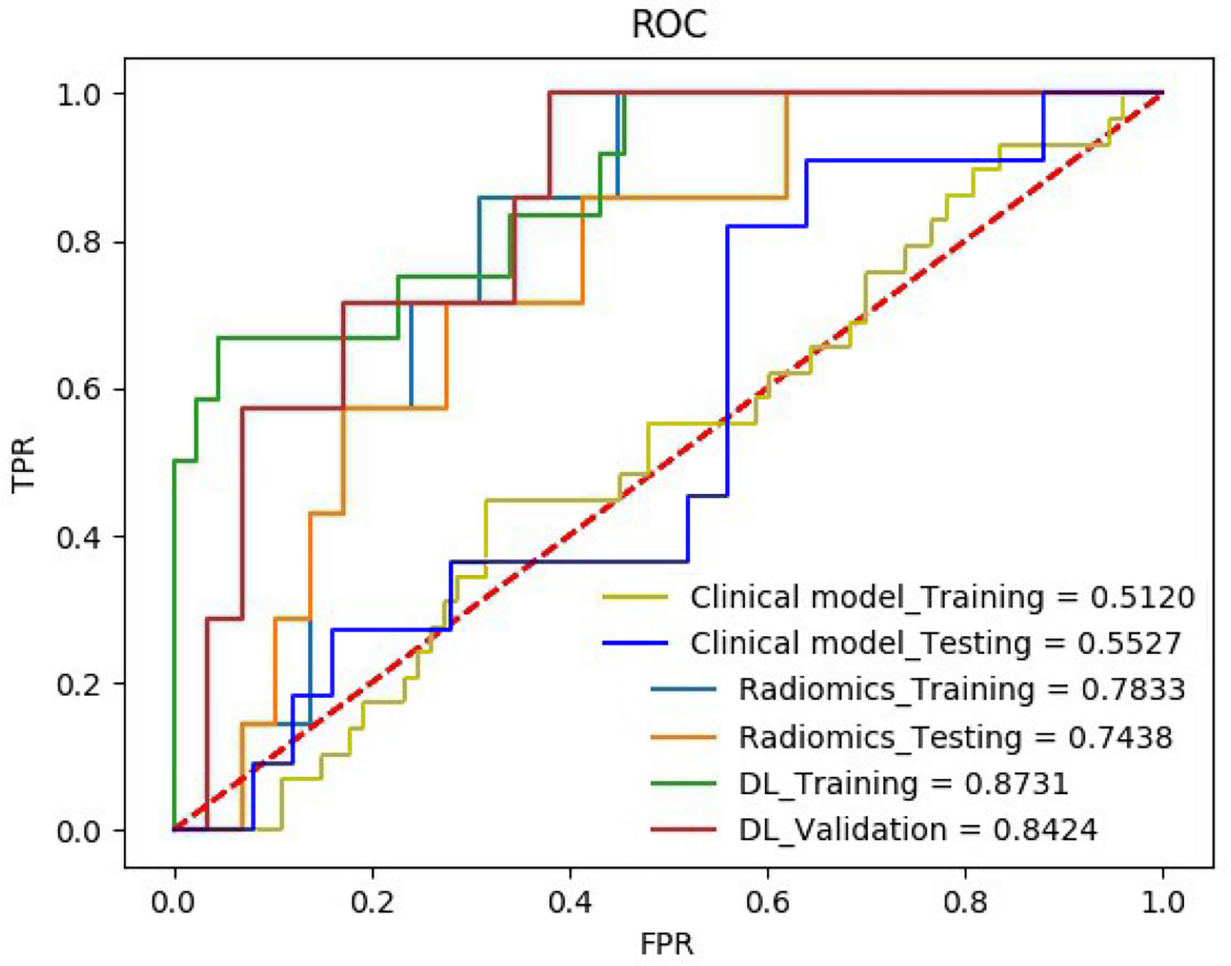
The receiver operating characteristic curve (ROC) of the HER2 on the training set and the testing set.

**Figure 6:**
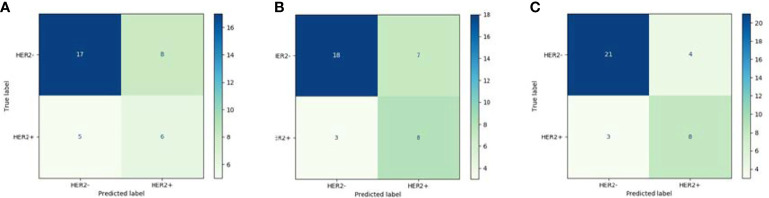
Confusion matrix: **(A)** clinical model; **(B)** radiomics model; **(C)** DL model.

The quantitative effectiveness is shown in **Table 2**, and the ROC curves are shown in **Figure 5**, which suggested that our proposed deep learning model significantly exceeded the clinical model (AUC = 0.55, accuracy = 68.52%, sensitivity = 52.94%, specificity = 75.68% in the training set; AUC = 0.51, accuracy = 63.89%, sensitivity = 54.55%, specificity = 68.02% in the testing set; *p <* 0.05) and the radiomics model (AUC = 0.78, accuracy = 71.29%, sensitivity = 55.88%, specificity = 78.38% in the training set; AUC = 0.74, accuracy = 72.22%, sensitivity = 72.72%, specificity = 72.00% in the testing set; *p <* 0.05). The confusion matrix shown in **Figure 6** reveals that the deep learning model achieved a lower confusion degree in comparison with the clinical model and radiomics model.

**Table 2:** Predictive performance of each model for HER2.

Prediction target	AUC	Accuracy	Sensitivity %	Specificity %	PPV %	NPV %
Clinical model training set	0.55	68.52%	52.94%	75.68%	50.01%	77.78
Clinical model validation set	0.51	63.89%	54.55%	68.02%	42.86%	77.27%
Radiomics model training set	0.78	71.29%	55.88%	78.38%	54.29%	79.45%
Radiomics model validation set	0.74	72.22%	72.72%	72.00%	53.33%	85.71%
Deep learning model training set	0.87	85.19%	73.53%	90.54%	78.12%	88.16%
Deep learning model validation set	0.84	80.56%	72.73%	84.00%	66.67%	87.5%

## Discussion

This work proposed a DenseNet-based deep learning model to predict HER2 expression in patients with breast cancer through preoperative non-invasive ultrasound images. The deep learning model was trained in the training cohort, which included 108 patients and was validated in the validated cohort, which included 36 patients. The proposed model was highly effective in the training cohort (accuracy = 85.79%, AUC = 0.87) and the validation cohort (accuracy = 80.56%, AUC = 0.84), exceeding the clinical model and radiomics model. The related tumor area representing HER2 expression status could be obtained by our model using the class activation map.

HER2 is a critical biomarker and its expression helps to make personalized treatments for breast cancer patients. Patients whose HER2 is positive should receive trastuzumab (marketed as Herceptin) which is effective only in cancers where HER2 is overexpressed (38). In clinical practice, IHC is widely used to evaluate the expression of HER2. HER2 expression is positive when the result of IHC is 3+, while HER2 is negative when IHC is 0 or 1+. In 2+ cases by IHC, fluorescence *in situ* hybridization (FISH) should be employed to confirm the final expression of HER2 (39). However, the IHC and FISH methods require an invasive approach to collect a sample and they are time-consuming. Due to the possibility of positional deviation, an invasive biopsy may fail, and wrong results may be obtained. The prediction of HER2 through preoperative ultrasound images using deep learning could compensate for the above lack.

Recently, the texture analysis-based radiomics method has been used for the diagnosis of the breast cancer (40). Before building a predictive model, ROI must be delineated by radiologists, and then texture features should be manually extracted. However, the delineation of the tumor boundary influences the extracted feature values. The deep learning model needs only an approximate rectangle ROI of the tumors rather than the accurate delineation of the boundaries compared to the conventional texture analysis-based radiomics which requires feature engineering (41). The proposed deep learning model with a multi-block structure and shortcut connections extracts features from raw image pixels to abstract maps without time-consuming handcrafted feature engineering. The model takes raw ultrasound images as input and then predicts HER2 expression.

Despite the promising effectiveness of the proposed deep learning method, this study has some limitations. First, the ultrasound images to build the model were collected from only one manufacture (Philips). Ultrasound signals emitted from different transducers produced by different manufacturers may lead to distinct image features. Hence, building a more general model should be considered in the future. Second, only one type of ultrasound image was used to build the model. In the future, the feature concatenation of convolutional operation in the neural network should be explored to build a two-branch model. Other types of images such as the color Doppler ultrasound or mammography may be considered for the two-modal model to increase the predictive performance. The combination of the deep learning-based tumor auto-detection and deep learning-based radiomics will be considered in the future to obtain a complete clinical diagnostic software.

## Conclusions

The above results demonstrate that features of pretreatment ultrasound images are related to HER2 expression. Our proposed deep learning model significantly exceeded the traditional texture analysis-based radiomics model. Our method without manual delineation of ROI is non-invasive, simple, and feasible.

## Data Availability Statement

The original contributions presented in the study are included in the article/supplementary material Further inquiries can be directed to the corresponding author.

## Ethics Statement

Written informed consent was obtained from the individual(s) for the publication of any potentially identifiable images or data included in this article.

## Author Contributions

ZX contributed to the study design, data acquisition, data analysis, data interpretation, software development, and manuscript drafting. QY contributed to the study design and data acquisition. ML contributed to the manuscript drafting. JG contributed to the manuscript drafting. CD contributed to the manuscript drafting. YC contributed to the data analysis and data interpretation. BL contributed to the study concept, study design, data acquisition, data analysis, data interpretation, and manuscript drafting. He is the PI of the study and oversaw the entirety of the project. All authors contributed to the article and approved the submitted version.

## Funding

This paper was supported in part by the National Key Research and Development Program of China (2016YFC0105106), in part by the academic promotion program of Shandong First Medical University (2019LJ004), in part by the National Natural Science Foundation of China (81530060), in part by the State’s Key Project of Research and Development Plan under Grant 2017YFC0109202 and Grant 2017YFA0104302, and in part by the National Natural Science Foundation under 61871117.

## Conflict of Interest

The authors declare that the research was conducted in the absence of any commercial or financial relationships that could be construed as a potential conflict of interest.

## Publisher’s Note

All claims expressed in this article are solely those of the authors and do not necessarily represent those of their affiliated organizations, or those of the publisher, the editors and the reviewers. Any product that may be evaluated in this article, or claim that may be made by its manufacturer, is not guaranteed or endorsed by the publisher.
