# Supplementary material for: Predicting HER2 Status in Breast Cancer on Ultrasound Images Using Deep Learning Method
Source: Front Oncol. 2022 Feb 16;12:829041. doi: 10.3389/fonc.2022.829041 (PMC8889619; doi:10.3389/fonc.2022.829041)
Supplement: Supplementary file 1 [file Table_1.pdf]

Supplementary materials

Supplementary Table 1. Architecture of the network model

Layers	Details
Convolution	kernel=[7, 7], stride=2
Max Pooling	kernel=[3, 3], stride=2
Dense Block 1	conv layer (kernel=[3, 3], stride=1) × 4, growth_k=12
Transition Layer 1	Batch Normalization Layer<br>ReLU Layer<br>Conv Layer (kernel=[1, 1], stride=1)<br>Average pooling (pool_size=[2, 2], stride=2)
Dense Block 2	Conv Layer (kernel=[3, 3], stride=1) × 4, growth_k=12
Transition Layer 2	Batch Normalization layer<br>ReLU Layer<br>Conv Layer (kernel=[1, 1], stride=1)<br>Average pooling (pool_size=[2, 2], stride=2)
Dense Block 3	Conv Layer (kernel=[3, 3], stride=1) × 32, growth_k=12
Transition Layer 3	Batch Normalization Layer<br>ReLU Layer<br>Conv Layer (kernel=[1, 1], stride=1)<br>Average pooling (pool_size=[2, 2], stride=2)
Classification Layer	Batch Normalization layer<br>ReLU layer<br>Global average pooling (stride=1)<br>Flatten Layer<br>Densely-connected Layer
