# Supplementary material for: Predicting HER2 Status in Breast Cancer on Ultrasound Images Using Deep Learning Method
Source: Front Oncol. 2022 Feb 16;12:829041. doi: 10.3389/fonc.2022.829041 (PMC8889619; doi:10.3389/fonc.2022.829041)
Supplement: Supplementary file 2 [file Table_2.pdf]

Supplementary Table 2. Parameters for Training Proposed Deep Learning Model

Parameters	Values
Mini-batch size	49
Loss function	Cross entropy
Optimization method	ADAM
Initial learning rate	10^{-4}
